# Diet in Health and Disease

**Published:** 1905-12

**Authors:** 


DIET IN HEALTH AND DISEASE. By Julius Friedenwald, M.D., Clinical Professor of Diseases of the Stomach in the College of Physicians and Surgeons, Baltimore; and John Ruhräh, M.D., Clinical Professor of Diseases of Children in the College of Physicians and Surgeons, Baltimore. Octavo volume of 689 pages. Philadelphia, New York, London: W. B. Saunders & Company, 1904. Cloth, $4.00 net.

This latest work on diet is practical and comprehensive, prepared to meet the needs of the general practitioner, medical student, hospital interne, and trained nurse. It contains a full account of foodstuffs, their uses and chemical compositions. Dietetic management in all diseases in which diet plays a part in treatment is carefully considered, the articles on diet in diseases of the digestive organs containing numerous diet-lists and explicit instructions for administering. The feeding of infants and children, of patients before and after anesthesia and surgical operations, and the latest methods for feeding after gastro-intestinal operations have never before been discussed with such practical detail. The subject of rectal enemata is given completely, with recipes and full instructions as to technic. Diet is considered in its relations to age, occupation and environment; and the beneficial results from the rest cure have been accorded prominent consideration. There is also a section on food adulteration and the resultant diseases. Withal, this is a work well worthy the reputation of its authors, and we most cheerfully recommend it.

